# Use of an Exposome Approach to Understand the Effects of Exposures From the Natural, Built, and Social Environments on Cardio-Vascular Disease Onset, Progression, and Outcomes

**DOI:** 10.3389/fpubh.2020.00379

**Published:** 2020-08-12

**Authors:** Paul D. Juarez, Darryl B. Hood, Min-Ae Song, Aramandla Ramesh

**Affiliations:** ^1^Meharry Medical College, Nashville, TN, United States; ^2^College of Public Health, The Ohio State University, Columbus, OH, United States

**Keywords:** exposome, cardiovascular disease, genomics, epigenomics, metabolomics, transcriptomics, proteomics, lipidomics

## Abstract

Obesity, diabetes, and hypertension have increased by epidemic proportions in recent years among African Americans in comparison to Whites resulting in significant adverse cardiovascular disease (CVD) disparities. Today, African Americans are 30% more likely to die of heart disease than Whites and twice as likely to have a stroke. The causes of these disparities are not yet well-understood. Improved methods for identifying underlying risk factors is a critical first step toward reducing Black:White CVD disparities. This article will focus on environmental exposures in the external environment and how they can lead to changes at the cellular, molecular, and organ level to increase the personal risk for CVD and lead to population level CVD racial disparities. The external environment is defined in three broad domains: natural (air, water, land), built (places you live, work, and play) and social (social, demographic, economic, and political). We will describe how environmental exposures in the natural, built, and social environments “get under the skin” to affect gene expression though epigenetic, pan-omics, and related mechanisms that lead to increased risk for adverse CVD health outcomes and population level disparities. We also will examine the important role of metabolomics, proteomics, transcriptomics, genomics, and epigenomics in understanding how exposures in the natural, built, and social environments lead to CVD disparities with implications for clinical, public health, and policy interventions. In this review, we apply an exposome approach to Black:White CVD racial disparities. The exposome is a measure of all the exposures of an individual across the life course and the relationship of those exposures to health effects. The exposome represents the totality of exogenous (external) and endogenous (internal) exposures from conception onwards, simultaneously distinguishing, characterizing, and quantifying etiologic, mediating, moderating, and co-occurring risk and protective factors and their relationship to disease. Specifically, it assesses the biological mechanisms and underlying pathways through which chemical and non-chemical environmental exposures are associated with CVD onset, progression and outcomes. The exposome is a promising approach for understanding the complex relationships among environment, behavior, biology, genetics, and disease phenotypes that underlie population level, Black: White CVD disparities.

## Introduction

The elimination of racial health disparities has been a stated national priority for over 30 years, yet little progress has been made in reducing them (1, 2). To date, while research on cardio-vascular disease (CVD) has identified a plethora of risk factors for CVD and CVD disparities our understanding of underlying causal mechanisms and pathways remains limited (3, 4) hindering our ability to develop effective prevention and treatment interventions.

### Fundamental Concepts, Issues, and Problems

The exposome was described by Wild in 2005 (5) as “life-course environmental exposures (including lifestyle factors), from the prenatal period onwards”. Yet, a conceptual framework and the tools needed to study the complex interactions between environment and health are still lacking (5).

### External Environment

Juarez (6) previously proposed an ontology of the external environmental which directly and indirectly affects health and health-related behavior as comprised of three broad domains: natural, built, and social environments (7). The natural environment was identified as being comprised of three elements in which people interact on a daily basis: air, water, and soil and can have negative or beneficial effects on health and health behavior. There is increasing awareness that green space, defined as “open, undeveloped land with natural vegetation” such as parks, forests, and may have salutogenic effects on health (8).

The built environment was described as including the characteristics of manmade entities of the communities in which we live, work and play, such as the neighborhoods in which we reside, homes in which we live, the buildings in which we work, and the transportation infrastructure that ties them together. The built environment is constructed largely of materials that are synthetic, chemically processed or treated, all of which can affect our health. Toxic chemicals that leak into the indoor air from the built environment are largely invisible and undetectable in our daily activities. The US Environmental Protection Agency estimates that people spend up to 90% of their time in buildings (9).

The degree to which neighborhood is vital to health and health behavior may vary considerably, depending on local public policy decisions. For instance, planning decisions that influence the location of supermarkets, fast-food eateries, farmers markets, and convenience stores can have profound effects on people's diets and their health (10). Inaccessible or non-existent sidewalks and bicycle or walking paths may contribute to sedentary habits. Additionally, a person's level of physical activity can be directly related to poor health outcomes such as obesity, cardiovascular disease, diabetes, and some types of cancer.

The social environment is comprised of the social, economic, and political conditions in which people live, work, and play. Social forces determine the conditions of daily life and are shaped by macro-level factors and include social norms, culture, social policies, economic conditions, and political systems (11). According to the Centers for Disease Control and Prevention, complex, integrated, and overlapping social structures and economic systems are responsible for most health inequities (12).

### Exposome

Environmental exposures are recognized as playing an important role in the etiology of many chronic diseases (13). Measuring the totality of environmental exposures that a person experiences across the life course has emerged as a recent field of study, now referred to as exposomics (14–16). An exposomics approach addresses the cumulative risks associated with interactions between multiple environmental exposures, biological perturbations, and epigenetic variations over time and over the life course (17–19).

There is an increasing recognition of the need for complex models to help us better understand how multiple and cumulative, environmental exposures affect chronic disease onset, progression, and outcomes at critical life stages, over the life course, and across generations (20, 21). However, much of the research on environmental exposures to date, has been largely limited to identifying relationships between individual chemical exposures and single health outcomes (3, 4, 22). The multiple mechanisms and pathways underlying CVD suggest the need for applying complex models that can account for the relationships between multifactorial, environmentally-induced, health-related symptoms, disease outcomes, and population level disparities (23, 24).

Exposomics provides an approach for understanding how environmental exposures where you live, work, and play can lead to CVD onset, progression, and outcomes. It combines a real world approach with cumulative risk models and big data tools capable of distinguishing exposure mechanisms and pathways underlying chronic diseases. It supports the use of computational and mathematical models and analytics capable of analyzing and modeling the complex relationships between multiple and cumulative exposures from the natural, built, and social environments, at different stages of life, with disease phenotypes, health outcomes, and population level disparities (25). Finally, the exposome provides a framework for understanding how biological mechanisms and exposure pathways at different stages of life and across the life course are involved in increasing and/or decreasing the risk for CVD.

### The Exposome and Cardio-Vascular Disease

**Figure 1:**
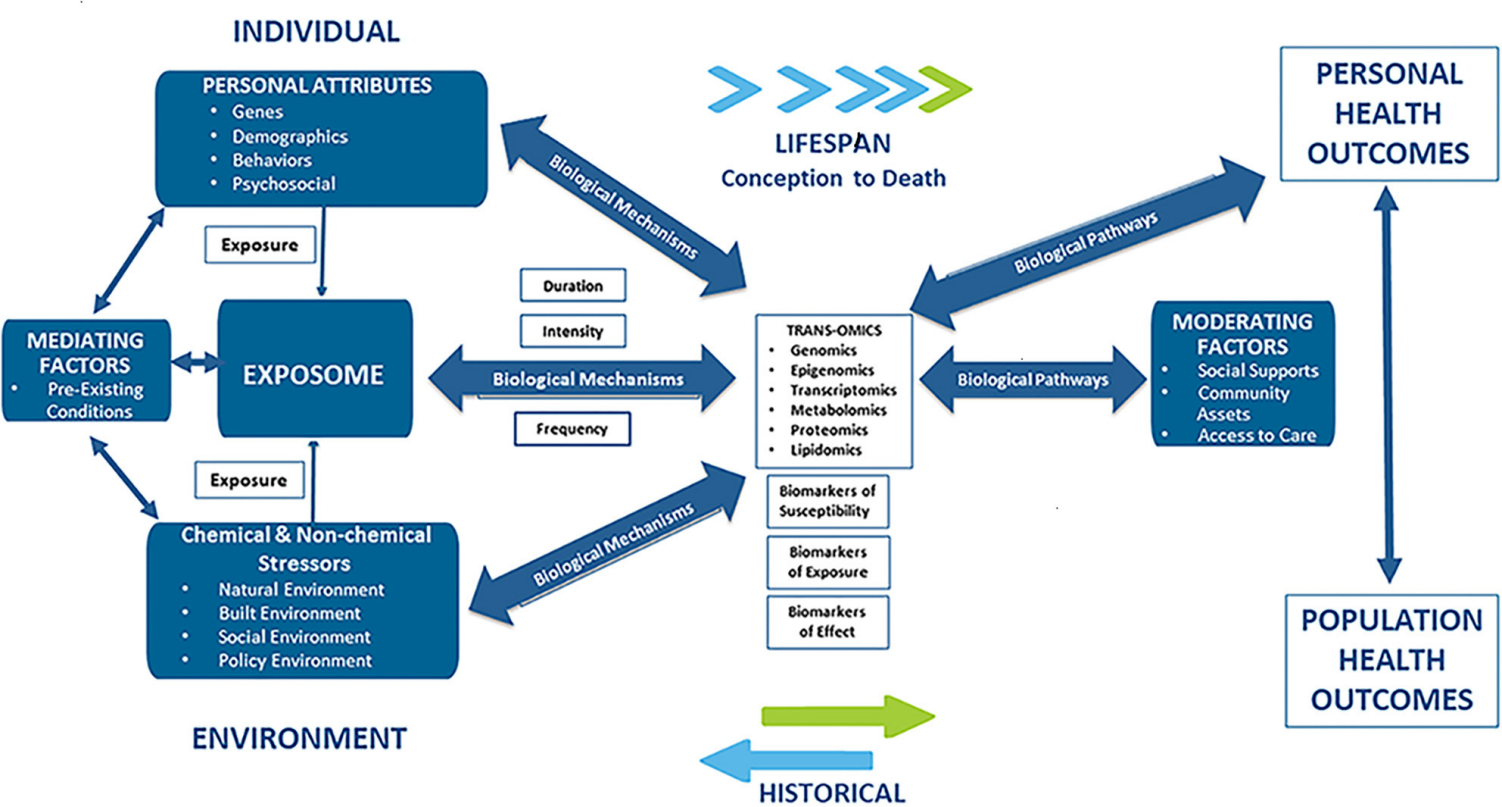
Applying an exposome approach to cardio-vascular disease onset, progression, and outcomes.

There is increasing evidence that racial disparities in CVD may be the result of “late manifestations of progressive vascular dysfunction initiated in early life” (26). This hypothesis points to multiple and cumulative exposures to stressful psychosocial and environmental forces as the underlying causes of CVD and CVD disparities. Additionally, the hypothesis states that adverse exposures that occur early in life actually enhance disease-promoting pathways impacting biologically-based, disease-susceptible phenotypes over the life course. This hypothesis is consistent with the Developmental Origins of Health and Disease (DOHaD) concept and the findings here provide for the formulation of new strategies for research as well as for interventions in the policy, public health, and clinical arenas (27). The various environmental, personal and social factors associated with CVD are schematically depicted in Figure 1.

Common CVD risk factors at the individual-level, include those that are non-modifiable and unique to individuals (i.e., age, race, gender, family history, and genetics), those that are modifiable (sedentary behavior (28–30), waist circumference, obesity, medication non-adherence (31, 32), alcohol intake (33, 34), exercise (35, 36), BMI, smoking (37, 38) occupation (39), and education (40); and others that are vascular-related (i.e., abdominal obesity, atherogenic dyslipidemia, raised blood pressure, insulin resistance, glucose intolerance, pro-inflammatory, and prothrombotic states). Other biological risk factors for CVD include high blood pressure (41), allostatic load (42–44) dyslipidemia, serum total cholesterol, decreased HDL cholesterol, triglycerides, fasting insulin (45), serum creatinine (45), serum uric acid (46) serum hsCRP (47), homocysteine, inflammation, hypertriglyceridemia, thrombosis, insulin resistance, serum lipids, and blood glucose (48–50), fibrinogen (50), and homocysteine (51).

Common CVD risk factors found in the natural environment include heavy metals (arsenic cadmium, lead, and mercury), pesticides, and solvents. Other risk factors found in the natural environment that contribute to CVD risk, include indoor pollution (second hand smoke, biomass fuels) (52), and outdoor air pollution comprised of particulate matter (PM^10^, PM^2.5^, ultrafine particles), complex mixtures of gases that include carbon monoxide (CO), diesel exhaust, nitrogen dioxide (NO_2_), ozone (O_3_), and sulfur dioxide (SO_2_) (53–59). Numerous epidemiological studies have found that ambient PM in air pollution is strongly associated with increased CVD morbidities and mortality, including atherosclerosis, cardiac arrhythmias, myocardial infarction (MI), diabetes, hypertension, ischemic stroke, and vascular dysfunction at relatively low concentrations (56, 60). There is growing evidence to suggest that particulate matter may help explain racial CVD disparities not accounted for by non-modifiable and social, demographic, and behavioral risk factors (55).

Common CVD risk factors found in the built environment include neighborhood level conditions (walkability, perceived/actual safety) and access to a healthy food environment (cost of healthy and unhealthy food and physical access assessed by density/availability of healthy or unhealthy stores/restaurants).

Common CVD risk factors associated with the social environment, include access to insurance and health care services (61–64), community stressors (65, 66), lack of trust in health care providers (64), population density, residential segregation (67, 68), socio-cultural beliefs and norms (car ownership, cultural influences), and availability of social supports (69). Public policies that have been identified as common CVD risk and/or protective factors, and as such, can have a direct or indirect outcome on CVD, including zoning ordinances regarding parks, foot paths and cycle ways, or policies that discourage driving or encourage use of public transit.

Using the exposome to model the contributions of multiple environmental exposures across the life course on CVD is in its formative stage. The exposome approach is presented in this article as a conceptual model for integrating exogenous, chemical and non-chemical exposures from the natural, built and social environments with data derived from internal, endogenous environment, using high-throughput “omics” techniques including genomics, epigenomics, transcriptomics, proteomics, metabolomics, and lipidomics, and clinical data (including diagnoses, clinical care, pharmacy, and health outcomes). Identification of biological mechanisms and causal pathways of CVD risk have important implications both for CVD risk assessment and stratification as well as for public health interventions, programs and policies (70).

**Figure 2:**
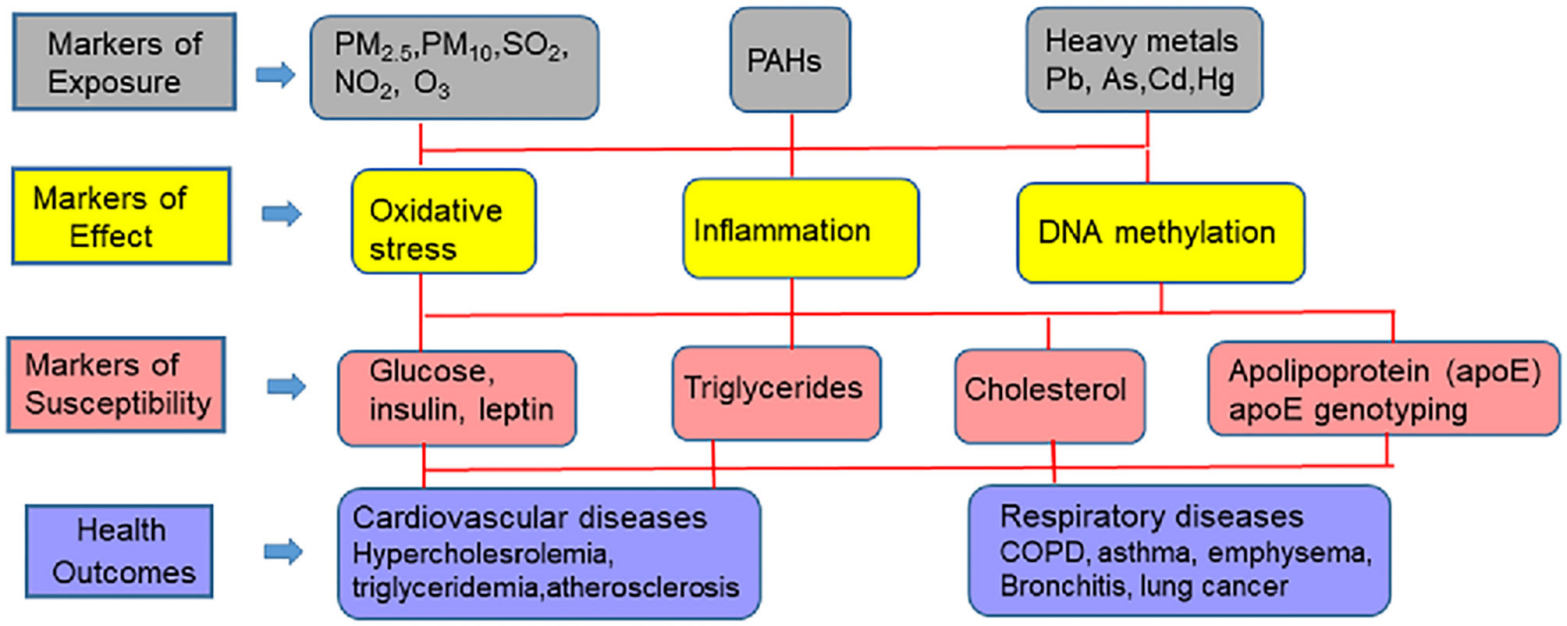
Multi-pollutant approach to biomarkers and CVD outcomes.

This review will cover progress that has been made in identifying the source of external environmental exposures and the mechanisms and pathways through which these exposures affect the internal environment, and together how they can be analyzed with biomarkers of exposure (environmental toxicants such as metals and hydrocarbons), biomarkers of effect (vascular and cellular adhesion molecule, c-reactive protein, β-defensin, interleukin-6, isoprostanes, glutathione, glutathione peroxidase 3, superoxide dismutase 3, and DNA methylation changes), and biomarkers of CVD susceptibility [glucose, insulin, low density- and high density lipoproteins, triglycerides, apoE genotyping (apoB, apoA1, and apoE)] to predict adverse CVD outcomes. The various pollutants and biomarkers associated with CVD are schematically presented in Figure 2. Implications for CVD racial disparities will be discussed.

### Biomarkers From the Perspective of Exposure Biology

As detailed in the aforementioned sections, environmental and life-style factors play a pivotal role in assessing the pathophysiology of CVD. In this regard, biomarkers play a key role in decoding the exposome as they are measures of characteristics that represent either normal or pathogenic biological processes. From a causal pathway perspective, the three major categories of biomarkers that assist in CVD assessment are given below.

#### Biomarkers of Disease Susceptibility

These markers are indicators of increased sensitivity to the effects of suspected environmental toxicants that can be measured in a biological sample or system. These markers provide information on what target systems are most vulnerable from the standpoint of a disease etiology. Biomarkers that fall under this category are concentrations of glucose, insulin, triglycerides, LDL and HDL cholesterol, and apolipoprotein (apo)B, apoAI, and apoE assays. These markers when measured in blood samples have shown a strong correlation with incidence of CVD in patients (71, 72). Also qualified under this category are genotyping studies that have identified participants that harbor apoE variants (73). These studies have helped us to assess which sub-groups of populations are at risk from CVD because of carrying apoE variants.

#### Biomarkers of Exposure

These markers provide information on the importance of various exposure pathways and risk. These markers enable direct measurement of toxicants of interest in the body from an accessible biological matrix such as blood, urine, tissues etc. Persons, who are occupationally exposed to environmental heavy metal toxicants such as Pb, Cd, As, Hg and PAHs, individually, or in mixtures are at increased risk for developing CVD and renal diseases (74).

#### Biomarkers of Effect

These markers are responses elicited as a result of interaction of an organism with a gamut of physical, social and environmental factors as mentioned in the earlier sections. The responses are measured at the level of tissue-, -organ and -whole organism function. Commonly measured biomarkers of CVD are F2 isoprostanes, Glutathione content (GT; total & oxidized), Glutathione peroxidase 3 assay (GP3), Superoxide dismutase 3 assay (SOD3), C-reactive protein (CRP), interleukin (IL) 6, 8 and 10, β- Defensin and Vascular Cell Adhesion Molecule1 (VCAM1). High levels of defensin, isoprostanes, VCAM1, CRP, IL, reduced GT, and SOD have been reported from patients, who are diagnosed with CVD (75–78).

## Current Research Gaps

Disruption of physiological homeostasis is one of the key steps in development of several diseases. Therefore, for understanding the pathophysiology of various diseases including CVD, a comprehensive assessment of various key biological events is necessary. Most studies of biomarkers used to date are hypothesis-driven and focus on a specific metabolite or protein or a toxicant isomer or congener. This approach is laborious and often focuses on biomarker of focus in its natural domain [biological site; (79)]. Molecular events such as gene expression and signaling events depend on the exposure scenario. In the case of complex diseases, they need to be categorized into sub-phenotypes on the basis of patient's genotype, which limit our capabilities in developing a broad and generalizable biomarker (80). To bridge this gap, researchers have leveraged the high-throughput technologies and computational biology to mine datasets for differential expressions at the level of genome, epigenome, transcriptome and proteome, collectively referred to as “omics” technologies. Integrative omics technologies has emerged as an approach with great promise for providing a more comprehensive view of biology, understanding disease mechanisms and pathways, and informing disease treatments (81).

## Developments in the Field

In this overview, we use an exposome approach to guide our discussion of how to combine diverse types of data and the utility of this approach for understanding CVD onset, progression, and outcomes. This section highlights developments in the field, and as such, references are representative studies and were not chosen on the basis of a systematic review, as such an undertaking is beyond the scope of this special issue. Inasmuch as possible, we have included objective evidence, regardless of whether the studies reported have been validated yet, as this review is expected to stimulate discussion among researchers on the role of omics in CVD.

### Biomarkers From the Perspective of Systems Biology and Development of Novel Biomarkers

In recent years, the use of key biological molecules for disease prediction and diagnosis have been harnessed differently. These molecules include metabolites and cellular macromolecules (proteins, lipids, and nucleic acids) which have varied functions including but not limited to cell signaling and immune modulation, serving as endogenous toxins, and environmental sensors. How these molecules synergistically influence organ function, rendering immunity, nutrient sensing and overall physiology play a key role from the perspective of systems biology (82).

An exposome approach is used to describe the integration of biological processes and how disruption of these processes by chemical and non-chemical stressors associated with environmental exposure, lifestyle, dietary habits, and occupation affects disease onset, progression, and outcomes. This type of comprehensive approach could reveal information used in delineating the site, level of toxicity, and mode of action of the response, and may be beneficial in defining adverse effect levels.

**Figure 3:**
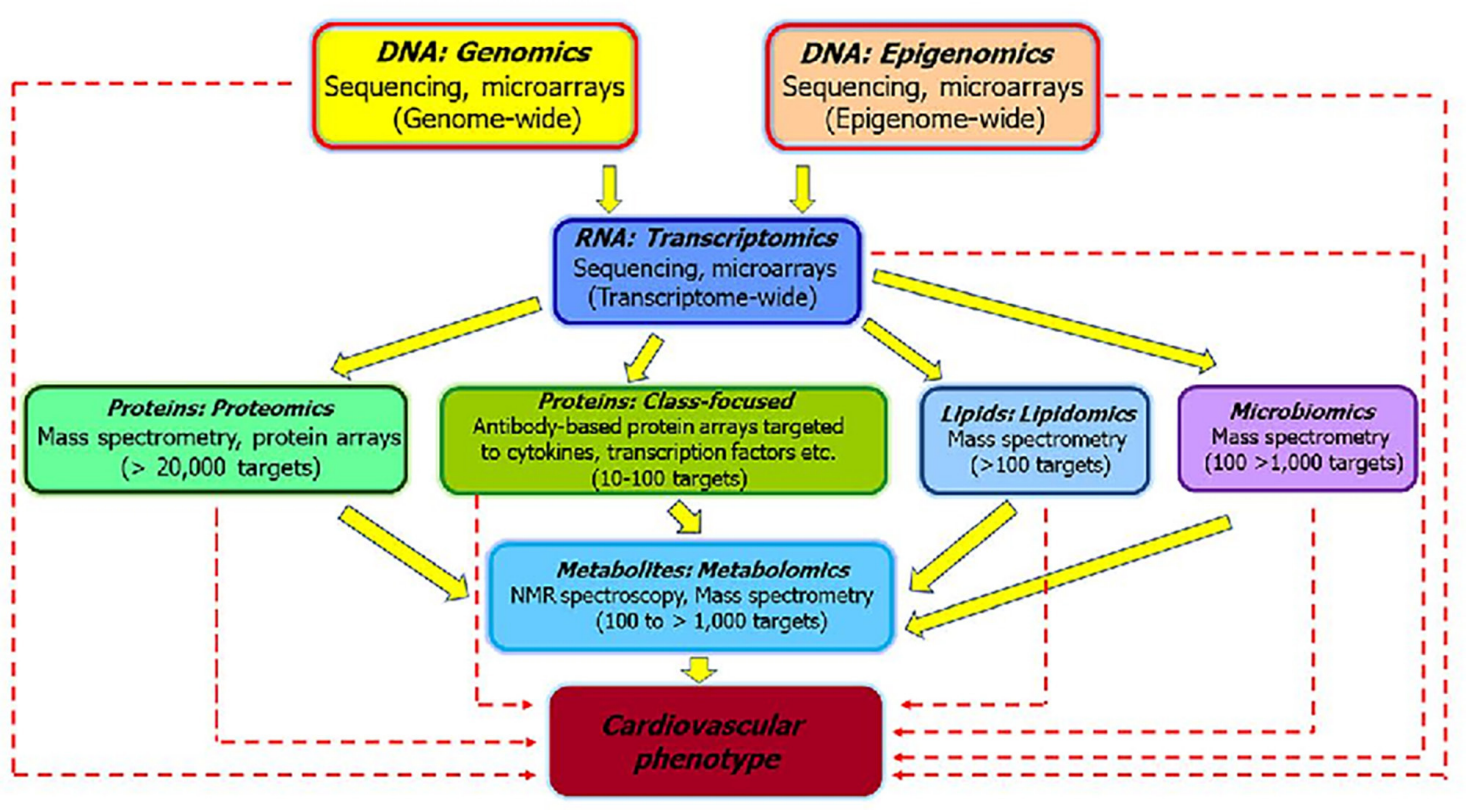
The various “omics” approaches widely used to elucidate the pathways that underlie pathophysiology of CVD and development of biomarkers [modified from Selley et al. (84)]. The sold arrows represent how different omics are interlinked and could be useful to investigate the mechanisms associated with CVD. The dashed arrows represent how individual omics could serve as biomarkers for distinguishing sub-phenotypes and helpful for clinical application of the findings.

The utilitarian value of systems biology is best understood by employing -omics approaches. These approaches will give rise to new biomarkers, which when standardized and validated could provide information on mode of action and dose-response relationships, inform and risk assessment purposes (83). The potential of omics approaches to elucidate mechanisms of toxicity (84) are schematically depicted in Figure 3. The various omics technologies employed for CVD risk prediction and the development of new biomarkers is detailed in the following sections.

### Genomics

Genomics, by definition, is the structure and action of the genome. In simple terms, it is the study of any individual's genes including interactions with each other and the individual's internal and external environment. In the past decade, there has been a quantum leap in knowledge about the human genome. As a result, thousands of genomes of persons from different ethnic backgrounds have been sequenced. Advances in technologies have helped us harvest genomic data for stratifying diseases into genetic and non-genetic categories. Additionally, the knowledge gained from genomics is useful for disease diagnosis and treatment.

Genomic biomarkers, including metabolomic, proteomic, lipidomic, epigenetic, and proteomic biomarkers are a complex topic. Even though the research literature shows hundreds of thousands of disease-associated molecular markers, very few of those are considered robust enough to be of clinical utility (85). The European Medical Agency coined the term “Genomic biomarker,” which is defined as “DNA or RNA characteristics that serve as an indicator of normal biologic processes, disease processes, and/or response to therapeutic or other interventions” (85) In this context, a genomic biomarker should reflect expression, function and regulation of genes. DNA characteristics include: single nucleotide polymorphisms (SNPs), DNA modifications, insertions, deletions, copy number variation and cytogenetic rearrangements. RNA characteristics include RNA sequence, RNA expression levels, RNA processing (splicing and editing) and microRNA levels (85).

#### Relevance of Genomics in Non-chemical Exposure-Induced CVD

Genomic studies are very relevant in non-toxicant exposure situations. The microarray-based gene profiling of peripheral blood mononuclear cells obtained from patients suffering from peripheral arterial disease (PAD) revealed upregulation of 40 genes and downregulation of 47 genes (86). The upregulated genes mediated immune response, inflammation, stress response, platelet activation, and aggregation while the downregulated genes are involved in transcriptional regulation. These findings could help in characterizing biomarkers for PAD. Other studies have shown altered gene and pathway expression linked to atherosclerosis. An analysis of atherosclerotic plaques showed dysregulation of several genes and differentially-expressed pathways linked to inflammation, especially leukocyte trafficking and signaling (87).

Genomic studies also have shown a close link between metabolic diseases and CVD. In patients with metabolic diseases such as diabetes, gene expression analyses revealed upregulation of 59 genes and down regulation of four genes (88). A great majority of the genes were related to endothelial dysfunction, which is the first step in vascular damage and may eventually lead to atherosclerosis and other cardiac disorders. A comprehensive review on relevance of GWAS and related measures analyzed across the genome in relation to CVD was provided by Ganesh et al. (89). The genetic underpinnings of coronary artery disease, stroke, hypertension, hypercholesterolemia, cardiomyopathies, arrhythmias, and aortic aneurysms were discussed in great detail in this review, and endorsed by the American Heart Association. The genomic biomarker data compiled from the several studies cited in Ganesh et al. (89) have therapeutic implications.

#### Relevance of Genomics in Chemical Exposure-Induced CVD

Cigarette smoke causes changes to the genetic material (DNA mutations) in smokers. These perturbations lead to smoking-related CVD. Smoking-related DNA damage in CVD has been reported to be manifested in different ways. The increase of micronuclei was correlated with development of atherosclerosis. Genomic instability (loss of heterozygosity and microsatellite instability) was found in atherosclerotic plaques. Additionally, DNA mismatch repair genes and nitric oxide synthase genes were found to be differentially expressed (90). Microarray analysis of genes isolated from the peripheral blood mononuclear cells showed 100 differentially expressed genes between cigarette smokers and moist snuff consumers. Genes from most cigarette smokers exhibited characteristic alterations in gene expression related to immune pathways (91). As smoking is strongly linked to CVD, studies like this help shortlist the possible biomarkers.

There are not many reports from human subjects that could link toxicant-induced gene expressions to CVD. In such cases, toxicant-gene signatures could be derived for widely distributed environmental toxicants from the Comparative Toxicogenomics Database (CTD; manually-curated information on toxicant X gene interactions and toxicant X gene X disease relationships). These toxicant-gene signatures could be linked with differentially expressed genes derived from the Gene Expression Omnibus Database (GEO; large gene expression data repository). This approach could be used for different diseases, including CVD (81, 85).

### Epigenomics

DNA methylation is the most well-known epigenetic regulator of gene expression without a change in the DNA sequence (92–94). DNA methylation is a biological process where a methyl group is added to deoxycytosine bases to form deoxymethylcytosine at mostly CpG sites (92). In the genome, regions with a high content of CpGs are known as CpG islands, which are found in ~ 50% of gene promoters, regulatory regions of gene expression (93–95). CpG island shores and shelves are sequences immediately flanking and up to two and four kilobases upstream and downstream of CpG islands, respectively (94). DNA methylation is accomplished with enzymes called DNA methyltransferases (DNMTs) (92, 94). These DNMTs transfer a methyl group from the methyl donor, S-adenosyl-L-methionine (SAM), to form 5-methyl cytosine (5mC) at CpGs (92–94). *De novo* DNMTs (DNMT3A and DNMT3B) adds the new methyl groups in cytosines that were not previously methylated (96). DNMT1 is responsible for the maintenance of methylation patterns during replication (97). DNA methylation is a dynamic and reversible biochemical process. What this means is that methyl groups at CpGs can be removed or demethylated and added. Reversing DNA methylation process is mediated by the ten-eleven translocation (TET) family enzymes-dependent oxidative pathways (98, 99).

Changes in DNA methylation have been associated with the biological and pathophysiological mechanisms of CVD (100–109). Frequently, studies show an association of DNA methylation of repetitive sequences such as LINE1 (>500,000 copies, accounting for about 20% of the genome (109) with various types of CVD (100, 102–104, 110). A study of cross-sectional and longitudinal analyses showed a significant association of LINE-1 hypomethylation (lowering methylation) with ischemic heart disease (Hazard ratio = 2.9, 95% CI = 1.3–6.2), suggesting hypomethylation of LINE-1 may be an indicator of risk for the disease (100). Similarly, myocardial infarction risk was significantly associated with hypomethylation of LINE-1 in men, but not in women (110). Consistently, an evidence-based systemic review article from 12,648 individuals across 31 studies concluded that there is an inverse association of global LINE1 methylation with CVD (103). In general, global hypomethylation is linked to a heightened risk of CVD. In contrast, a cross-sectional study of 420 Japanese subjects showed significant positive associations of blood LINE-1 methylation with the prevalence of dyslipidemia (102), which is a known risk factor for CVD (104). This conflicting finding may be explained by the difference in LINE-1 DNA methylation with other factors associated with race/ethnicity (111).

With advances in technologies for DNA methylation profiling, molecular epidemiologic studies show an association of genome-wide DNA methylation alterations with CVD (103, 105–108). A meta-analysis from 11,461 individuals identified seven differentially-methylated CpGs, annotated to DTX3L-PARP3, NLRC5, and ABO (105). The first two genes were negatively associated with circulating inflammatory cytokine, TNF-α levels (105), indicating the potential of DNA methylation for therapeutic applications. A systemic review found 34 CpGs linked to CVD, including methylation at F2RL3, ABCA1, KCNQ1, and C1QL4 (103).

In two large cohort studies, the Women's Health Initiative study (*n* = 2,129) and the Framingham Heart Study (*n* = 2,726), altered DNA methylation in three genes (SLC9A1, SLC1A5, and TNRC6C) was significantly associated with CVD risk (106). Further, these studies found a causal impact of SLC1A5's methylation on CVD (106). Gene-specific promoter DNA methylation was altered in the blood of patients with coronary artery disease, the most common CVD (107), compared to controls. Another recent study identified over 60,000 CpGs to be differential methylation in ischemic from non-ischemic cardiomyopathy (108). By integration of genome-wide methylation data and gene expression profiling, these studies found corresponding genes were enriched in oxidative metabolism, anaerobic glycolysis, and altered cellular remodeling (108).

#### Relevance of Epigenomics in Non-chemical Exposure-Induced CVD

While chemical exposures are the most widely investigated environmental factor linked to DNA methylation, non-chemical exposures in the social environment also been found to impact DNA methylation (112–117). Furthermore, neighborhood environmental factors such as socioeconomic position or socioeconomic status are known to be CVD risk factors (118, 119). Thus, it is plausible that DNA methylation may play a mediator of the association between social exposure and CVD in which DNA methylation alters stress and inflammation-related biological pathways (120).

A study of 1,226 individuals of the Multi-Ethnic Study of Atherosclerosis (MESA) study identified several differentially methylated CpGs associated with multi-dimensions of neighborhood characteristics (121). That study reported socioeconomic disadvantages were significantly associated with stress (CRF, SLC6A4) and inflammatory-related genes [F8, TLR1. (121)] Neighborhood social environment measured by aesthetic quality, safety, and social cohesion, also was found to be associated with stress (AVP, BDNF, FKBP5, SLC6A4) and inflammation-related genes [CCL1, CD1D, F8, KLRG1, NLRP12, SLAMF7, TLR1 (121)]. A study of a Mexican-American birth cohort (*n* = 241) found a positive association of LINE-1 with living in the most impoverished neighborhoods, but no association was found with socioeconomic status (122). Other studies have shown that maternal social resources may alter the imprinted MEG3 methylation of offspring, as measured in newborns' cord blood (123). Given that maternally expressed MEG3 is a long-noncoding RNA playing a role in angiogenesis and diabetes-related microvascular dysfunction (124), altered MEG3 methylation under the prenatal condition may relate to adult health such as CVD.

#### Relevance of Epigenomics in Chemical Exposure-Induced CVD

DNA methylation also provides the potential for a biomarker of exposure to reflect environmental and lifestyle risk factors and a biomarker of effect to demonstrate susceptibility to exposure-associated diseases. Although DNA methylation is structurally stable by a covalent modification (123), it can be modified by environmental exposure that leads to disease, including CVD or increased risk for developing CVD (120–122, 125–136).

Numerous chemical and non-chemical exposures also have been associated with both altered global and gene-specific DNA methylation. The exposure triggers inflammatory gene activation and oxidative stress leading to various types of CVD. Global hypomethylation (LINE-1 or ALU) or gene-specific promoter hypermethylation have been associated with chemical exposure such as air pollution [ambient particulate matter (PM)] (109, 111–114, 125, 137–139), heavy metals (140–143), and tobacco smoke (144, 145), as well as non-chemical exposures (109, 112–114, 125, 146).

Air pollution has been associated with CVD and DNA methylation suggesting an underlying biological process linking air pollution and CVD. A cohort study of 718 elderly individuals showed a negative relationship between PM_2.5_ and LINE-1 methylation [(β = −0.13, 95% CI, *P* = 0.001 (125)]. Another study showed PM-induced hypomethylation of ALU and its association with increased diastolic blood pressure (124). In a recent large genome-wide association study (*n* = 8,397) in the multi-racial/ethnic U.S. populations, three PM-sensitive CpGs (MATN4, ARPP2, and CFTR) were found annotated to a neurological, pulmonary, endocrine, or cardiovascular disease-related gene (126). However, the finding was not replicated in an independent dataset, comprised of white, European men and women living in Germany, which may be due to the differences in environmental diversity and/or other factors associated with race/ethnicity (126).

Coagulation and inflammation are pathogenetic mechanisms related to CVD (60). Methylation levels at a coagulation factor III (F3) gene have been found to be significantly associated with black carbon concentration while a significant decrease in mediated effects of sulfate and ozone on ICAM-1 protein, a putative early CVD risk marker have been found (127).

Heavy metals such as cadmium and arsenic also have been linked to increased risk for CVD and cardiovascular mortality (147, 148). *Cadmium*: An experimental animal study revealed that lowering DNA methylation by the DNA methylation inhibitor attenuates cadmium-induced cardiac contractile (128). A genome-wide DNA methylation profile of 43 women revealed associations of promoter methylation of GSTM and COL1A2 with mercury exposure and lead, respectively (129). A strong association of blood cadmium levels with MEG3 hypermethylation was observed in African American women (β = 3.52, *P* = 0.01) compared to those in White women (β = 1.24, *P* = 0.56) or Hispanic women [β = 1.18, *P* = 0.34 (130)]. *Arsenic*: Global hypomethylation measured by LINE-1 and ALU has been associated with increased arsenic exposure (131). Exposure to arsenic *in utero* has been found to influence over 550 CpGs in cord blood with enriched genes linked to CVD (30) while plasma folate, a methyl donor nutrient, also has been found to act as an effect modifier of the association (131).

Tobacco smoking has been identified as the primary risk factor for CVD causing one of every four deaths from CVD (149). A study of 934 individuals of the community-based Multi-Ethnic Study of Atherosclerosis (MESA) revealed 176 CpGs associated with urinary cotinine levels (133). A recent study of 485 carotid endarterectomy patients identified four CpGs corresponding to AHRR and ITPK1. Another large study (*n* = 16 cohorts, *n* = 15,907) found 2,623 smoking-linked CpGs with some genes related to coronary heart disease (enrichment *P* = 0.0028) (134). Similarly, differential methylation in 211 CpGs was found among individuals with a history of myocardial infarction, and about 20% of corresponding genes are related to cardiovascular function (135). Three CpGs located in ZFHX3 and SMARCA4 were associated with myocardial infarction, even after adjusting for CVD risk factors (136). Interestingly, the methylation levels of these CpGs seemed to be affected by single nucleotide polymorphisms in these CpGs, indicating a cross-talk between genetic and epigenetic factors (136).

### Transcriptomics

In this section, we focus on how transcriptomics might provide new opportunities for discovery that may lead to the next generation of therapeutics and analytics for altering and predicting negative cardiovascular disease trajectories. The term transcriptomics refers to the collective methodology applied to the study of RNA and of the RNA group as the transcriptome. The most studied RNA group is represented by the messenger RNAs (mRNAs) didactically defined as ribonucleotide sequences that are complementary to the coding strand of the genomic DNA (150). For an excellent review, please see (150).

#### Relevance of Transcriptomics in Non-chemical Exposure-Induced CVD

For the purposes of this review, we will focus on long-noncoding RNAs (lncRNAs), an RNA group that has stimulated much discussion over the past two decades as potential biomarkers. This heterogeneous group of lncRNAs are responsible for the regulation of gene expression at both the transcriptional and post transcriptional levels (151). lncRNAs were first characterized in breast cancer studies and have only recently been applied to cardiometabolic diseases (152). A seminal study demonstrated that the myocardial transcriptome is dynamically regulated in advanced heart failure and that lncRNAs expression profiling can be used to discern adverse outcome pathways in compromised hearts (153). Subsequent studies have utilized microarray technology to interrogate plasma samples in patients that had either undergone (or not) left ventricular remodeling after myocardial infarction. These studies identified a mitochondrial lncRNA LIPCAR as a potential biomarker of developmental processes in myocardial infarction patients, with additional association with cardiovascular death, independent of other risk factors (154). These studies and others laid the foundation for currently accepted hypotheses that lncRNAs have significant regulatory roles in cardiac pathophysiology and a potential prognostic indicator of cardiovascular disease development (155).

When discussing the myriad of diseases that fall under the broad cardiovascular designation of acute myocardial infarction (AMI), it is particularly relevant to note the disproportionality of this diagnosis in susceptible and vulnerable populations where there is hypothesized to be a place-based, disparate health outcome (156). AMI is a sudden cardiovascular event that stimulates remodeling of the myometrium and often leads to heart failure particularly in susceptible and vulnerable populations (149). In order to mitigate the observed mortality in this population, a therapeutic strategy is needed with a facile diagnosis that is both specific and sensitive. Just as various proteins are released subsequent to acute myocardial infarction (such as creatinine kinase and troponin C), as reviewed by Viereck et al. (157), it is likely that heart tissue damage causes an additional release of ncRNAs analogous to the release of proteins. As in the documented cases for established biomarkers such as circulating miRNAs, lncRNAs, and most probably, circular lncRNAs present as viable prospects that likely reflect cardiac myometrial injury, potential involvement of other organ systems, and cumulative cardiometabolic trajectory of the patient (158). lncRNA with strong diagnostic and prognostic relevance are presented in Table 1 (adapted from with permission from the authors; Viereck et al. (157).

**Table 1:** LncRNA biomarker in cardiovascular disease and injury.

**Disease**	**LncRNA**	**Regulation**	**Purpose**	**Normalization**	**Controls**	**Cases**	**Event Rate** **(Follow-Up Time)**
AMI	UCA1	Biphasic	Diagnostic	U6 snRNA	15	49 AMI	
	LIPCAR	Biphasic	Diagnostic/prognostic: death or LV remodeling	Cell-miR-39		246 AMI, 344 HF	LV remodeling: 38.5% (1 y)
	MYHEART	↑	Diagnostic	5S rRNA	28	47 AMI	
CAD	CoroMarker	↑	Diagnostic	B-actin	20	20 CAD	
	CoroMarker	↑	Diagnostic	Internal control	187	221 CAD	
	LncPPARδ	↑	Diagnostic	Gapdh	171^*†^	211 CAD	
HEM	Uc004cov.4	↑	Diagnostic	Cell-miR-39	26	28 HNCM, 57 HOCM	
	Uc022bqu.1	↑	Diagnostic	Cell-miR-39	26	28 HNCM, 57 HOCM	
HF	SENCR	↑	Diagnostic	Cell-miR-39	12	78 Type 2 diabetes mellitus	

*† *Indicates if controls have been matched to age and sex*.

*AMI, acute myocardial infarction; CAD, coronary artery disease; CCS, case-control study; HCM, hypertrophic cardio-myopathy; HF, heart failure; HNCM, hypertrophic nonobstructive cardiomyopathy; HOCM, hypertrophic obstructive cardiomyopathy; LIPCAR, long intergenic ncRNA predicting cardiac remodeling; lncRNA, long noncoding RNA; LV, left ventricle; MYHEART, myosin heavy-chain–associated RNA transcript; N/A, not available; rRNA, ribosomal RNA; SENCR, smooth muscle and endothelial cell–enriched migration/differentiation–associated long noncoding RNA; snRNA, small nuclear RNA; and UCA1, urothelial carcinoma-associated 1. Reprinted in partial form with permission from the author*.

This study reported that global lncRNA profiling from plasma was conducted in patients with left ventricular cardiac remodeling after AMI enumerated a mitochondrial transcript, referred to as long intergenic ncRNA predicting cardiac remodeling (LIPCAR), long (154). This study demonstrated the predictive power of LIPCAR as a biomarker and giving promise to this class of RNSa. It previously has been established that, MYHEART (myosin heavy-chain–associated RNA transcript), a lncRNA that protects the heart from hypertrophic remodeling, was upregulated in AMI patients as compared with control patients and has a positive correlation with a well-known cardiac injury marker UCA1 (urothelial carcinoma-associated 1) that was downregulated shortly after AMI development (159).

#### Relevance of Transcriptomics in Chemical Exposure-Induced CVD

There are limited transcriptomic biomarker studies in human subjects exposed to toxicants. Most of the studies pertaining to development of transcriptomic biomarkers have been carried out in animal models, which we have not addressed here as it is beyond the scope of this review. In complex exposure scenarios such as exposure to cigarette smoke or particulate matter, which contain a variety of toxicants, exposure assessment on the basis of transcriptomics is rather difficult. This is because multiple toxicants in the mixture elicit expression of the same marker making interpretations complicated (160).

In one human study of toxic exposures, Charlesworth et al. (161) reported the genome-wide quantitative transcriptional profiles from lymphocytes of non-smokers and smokers. After adjusting for residual genetic effects, 323 unique genes were identified whose expression levels were significantly correlated with smoking behavior. Most of the genes were linked to pathways associated with immune response, cell death, natural killer cell signaling, and toxicant metabolism. Some of the genes associated with the affected pathways were linked to CVD. Additionally, some genes that were slated for testing in non-chemical exposure settings could also be used in a variety of chemical exposure settings.

### Proteomics

Proteomics is the study of large-scale expression, function and interaction of proteins of an individual in healthy or disease states (162). Environmental toxicants, drugs, diet etc. react with proteins in several ways, including the formation of adducts, alteration of phosphorylation status (phorbol esters), alteration of thiols (ROS), and conversion of side chains to aldehyde or ketone groups (ROS) [reviewed in Dowling and Shehan (163)]. All these interactions lead to change in specific levels of proteins, which could be used as biomarkers. In regard to CVD, the perturbations to cellular protein homeostasis (proteostasis) caused by diet, obesity and stress (164) and environmentally-induced ‘wear and tear’ (165) leads to senescence of cardiac cells. For characterizing markers associated with aging of cardiac cells and CVD, the power of recent technological developments in the field of proteomics should shortly allow for the identification and validation of hitherto unknown biomarkers.

#### Relevance of Proteomics in Non-chemical Exposure-Induced CVD

In addition to traditional biomarkers used for CVD such as c-reactive protein and fibrinogen (166), there are a plethora of proteins associated with cardiovascular physiology that could serve as biomarkers in a non-chemical exposure situation. Studies conducted with heart muscle cells have revealed key pathway-associated proteins that could serve as markers of non-chemical exposures. The protein kinase C (PKC) is one of the key molecules involved in cell signaling through endothelial nitric oxide synthase (NOS) and Akt signaling that has been suggested as a marker (167). Similarly, proteins involved in mitochondrial signaling in the myocardium of different cardiac phenotypes are reflected in stress response and energy metabolism in mitochondria. Abnormalities in profiles of elastin and collagen involved in arterial wall dilation are other potential markers of vascular injury (166). The inhibition of leucocyte adhesion molecule and caveolins are indicative of endothelial dysfunction and could serve as markers for atherosclerotic disease (166). Troponin, associated with cardiac muscle contraction is another potential biomarker for myocardial injury (166).

In the Framingham Heart Study, a discovery proteomic platform was used to target 85 circulating protein biomarkers and identify risk for cardiovascular disorders (168). The researchers shortlisted key biomarkers on the basis of their association with atherosclerotic CVD. Among the biomarkers reported, leptin and N-terminal pro-b-type natriuretic peptide were associated with incident heart failure. Cardiovascular and cardiometabolic mortalities were associated with arabinogalactan protein 1 (AGP1), C-type lectin domain family 3 member B (CLEC3B; tetranectin), cystatin-C, kallikrein B1 (KLKB1), insulin-like growth factor 1, N-terminal pro-b-type natriuretic peptide, peripheral myelin protein 2 (PMP2), soluble receptor for advanced glycation end products (sRAGE), and uncarboxylated matrix Gla protein (UCMGP) (168). Studies in a multi-ethnic cohort revealed a positive association between glycosylated acute phase protein (GlycA: a novel biomarker of systemic inflammation) and suboptimal cardiovascular health (169). Study results indicated that GlycA, derived from glycosylation of major acute inflammatory proteins and a stable biomarker of systemic inflammation compared to other markers (167, 170) could be deployed for biomonitoring populations that have different lifestyles (dietary preferences, smoking etc.) or experience chemical exposures (occupational workers).

#### Relevance of Proteomics in Chemical Exposure-Induced CVD

Persons who are occupationally exposed to environmental heavy metal toxicants such as Pb, Cd and As, individually or in mixtures, are at increased risk for developing CVD and renal diseases (166). Poreba et al. (171) reported that occupational exposure to lead causes cardiac dysfunction and hypertension and suggested the use of cystatin C in serum as a prognostic marker for CVD.

In a population-based proteomics study, Borné et al. (172) examined the association between blood cadmium levels and 88 potential protein biomarkers for CVD. Their findings revealed that tumor necrosis factor receptor-2, matrix metalloproteinase-12, cathepsin L1, urokinase plasminogen activator receptor, and chemokine (C-X3-C motif) ligand-1 proteins were associated with blood cadmium in non- smokers and long-term former smokers and were significantly associated with incidence of CVD. These marker proteins suggest likely pathways by which cadmium exposure contributes to the development and promotion of CVD.

Similarly, chimney sweeps, who are occupationally exposed to particulate matter and PAHs, were reported to have CVD-related proteins [protein-glutamine gamma-glutamyltransferase 2 (TGM2), glyoxalase I (GLO1), NF-kappa-B essential modulator (NEMO), follistatin (FS), prointerleukin-16 (IL-16), and heat shock protein beta-1 (HSP 27)] in their sera. These serum protein markers also showed a positive association with the monohydroxylated metabolites of PAHs (173). Some of these proteins were correlated with homocysteine and cholesterol. Elevated level of homocysteine has been previously established as a risk factor for CVD by inducing endothelial damage and causing vasoconstriction by reducing the levels of nitric oxide, which is a vasodilator (174). Exposure to PAHs also may have induced oxidative stress in chimney sweeps, which could lead to endothelial dysfunction and inflammation resulting in atherosclerosis and hypertension (175). The increased expression of GLO1, NEMO, FS, and HSP 27 in chimney sweeps could be a compensatory response against PAH-induced oxidative damage (173).

In another occupational exposure study involving coke oven workers, blood concentrations of serum amyloid A (SAA), an acute phase inflammatory marker protein, was correlated to 1 hydroxypyrene, a biomarker of long-term PAH exposure (176). Since SAA contributes to atherosclerotic plaque formation (177), it could be used as a predictive biomarker of CVD risk in humans (178).

### Metabolomics

Metabolomics research which comprise both exogenous and endogenous metabolites has gained momentum in recent years. Metabolomics provide a comprehensive picture of the metabolite concentrations in the body in response to any kind of pathophysiological stimuli or genetic modifications (179, 180). Exogenous metabolites are related to diet and medicine intake whereas endogenous metabolites are produced as a result of several metabolic processes that occur in either a healthy or diseased state. Both categories of metabolites serve as novel biomarkers: the exogenous for dietary habits and therapeutic compliance and the endogenous for disease processes (180). Metabolomics provides an ideal platform for integrating genomic, lipidomic, epigenetic, transcriptomic and proteomic variations in an individual, notwithstanding the inherited genetic variations among individuals. It also is responsive to environmental exposures (toxicants), dietary and lifestyle (physical activity, smoking etc.) factors (181).

#### Relevance of Metabolomics in Non-chemical Exposure-Induced CVD

Thirteen identified—and four unidentified metabolites associated with altered lipid metabolism were recorded among White and African-American participants of the Bogalusa Heart Study (182). An association of metabolites with lipids provide insight into mechanisms that underlie lipid regulation and have implications for identifying new biomarkers for dyslipidemia, a major risk factor for cardiovascular disease. In the BHS cohort, five metabolites were reported to be associated with left ventricular diastolic dysfunction. Study findings also implicated the biological pathways underlying serum metabolome associated with heart failure (183). Another study revealed dysregulated metabolism in patients with coronary heart disease (CHD). The metabolic profile was indicative of reduced phospholipid metabolism and increased monoglyceride and abnormal fatty acid metabolism. The metabolites linked to the altered metabolic pathways are potential plasma biomarkers for diagnosis of CHD (184).

#### Relevance of Metabolomics in Chemical Exposure-Induced CVD

In recent years, high-resolution metabolomics (HRM) has been used to relate internal exposure of participants to complex traffic-related air pollution mixtures (185). In one study, plasma and saliva samples collected from the participants and subjected to HRM revealed differences in endogenous signaling processes that were related to oxidative stress, inflammation, nucleic acid damage, and repair. Such studies underscore the importance of untargeted HRM in the development of metabolic biomarkers for exposure and response to pollution arising from particulate matter.

The metabolomics approach has been successfully employed for identifying metabolic disorders associated with PM_2.5_ in human lung cells (186). Exposure to PM_2.5_ was found to alter sphingolipid metabolism and expression of key enzymes involved in this process. Additionally, PM_2.5_ exposure was found to induce the secretion of pro-inflammatory cytokines, which, along with the altered endogenous metabolites could serve as biomarkers of effect at the metabolic level.

An exhaustive review by Bonvallot et al. (187) revealed that in addition to PM_2.5_, diverse groups of toxicants such as PAHs, heavy metals, organochlorine compounds, and plasticizers affect common metabolic pathways. Ambient and occupational exposures to these toxicants lead to disruption of signaling pathways associated with inflammation and oxidative stress, which are drivers of atherosclerosis and metabolic disorders (188). However, to apply biomarkers derived from metabolomics on a population level, more research is needed to validate these biomarkers in different groups of similarly exposed population (189).

### Lipidomics

Lipidomics, an offshoot of metabolomics, denotes the study of global lipid and lipid derivative profiles (separation and identification) in biological fluids. Similar to metabolomics, lipidomics entails a high-throughput approach. Lipidomics serves to identify the involvement of lipids in inflammatory processes, immune system regulation, cell signaling, and onset of diseases. Knowledge gained from altered lipid profiles could be used to introduce lipid mediators (drugs to lower the lipid levels) in different inflammatory and metabolic conditions (190). A lipidomics approach is very powerful as it enables identification of hundreds of lipid species that could be tied to cardiovascular risk, including obesity, and diabetes diseases in a population setting (191).

#### Relevance of Lipidomics in Non-chemical Exposure-Induced CVD

In individuals who are not exposed to toxicants, but are at risk for CVD due to their lifestyle habits (diet and smoking), lipid profiles in plasma and erythrocyte membranes are potential biomarkers. Non-alcoholic fatty liver disease (NAFLD) share common features such as inflammation and excess lipid accumulation with other cardiometabolic diseases and could serve as disease markers (192). In a study of adolescent CVD population-based samples, Syme et al. (193) found several novel glycerophosphocoline (a glycerolipid) subtypes associated with CVD risk factors, including excess visceral fat, fasting insulin, and triacylglycerol levels. Plasma samples obtained from a population-based study found cholesterol esters (CEs), lysophosphatidylcholines, phosphatidylcholines, phosphatidyl-ethanolamines (PEs), sphingomyelins, and triacylglycerols (TAGs) were associated with CVD. Of these, TAGs and CE have the strongest predictive value for CVD and offer promise as new biomarkers that could outperform the lipid classes that currently are being used (194). Lipidomic profiles associated with high LDL-C and triglycerides also have been identified as biomarkers common to both familial hyperlipidemia and population-based hyperlipidemia indicating their robustness in providing molecular lipid signatures for coronary artery disease (195).

#### Relevance of Lipidomics in Chemical Exposure-Induced CVD

In a study of PM_2.5_ -induced cytotoxicity to human lung cells, nineteen lipids were found to be increased and three decreased as a result of PM exposure, suggesting lipotoxicity. In a recent epidemiological study by Zhang et al. (196), PM_2.5_ exposure was found to cause inflammation and alterations in lipids associated with atherosclerosis. The findings of this study suggest that inflammation promoted plaque accumulation that was initiated through lipid dysregulation.

In a cohort of patients with chronic obstructive pulmonary lung disease (COPD), and suspected cardiac issues, an increase in glycerol (phospho) lipids including triglycerols, decrease in ω-3 polyunsaturated fatty acids, imbalance in eicosanoids and decrease in hydroxyoctadecadienoic acids, was noted in smokers (197). Since these lipids also play an important role in CVD, alterations in observed lipid profiles could be expected in people suffering from CVD. Lipids from exhaled breath condensate (EBC) collected could also serve as biomarkers to distinguish between healthy people and those who are at risk for lung and heart diseases due to their smoking habits. In a study using EBC, products of major arachidonic acid lipoxygenation and cyclooxygenation pathways were found to be elevated in smokers. As these compounds are markers of inflammation, their concentrations in serum are indicative of lung and cardiovascular health issues (198).

Aside from epidemiologic studies, the utility of lipids as biomarkers have been explored in human cell lines exposed to toxicant mixtures such as cadmium and benzo(a)pyrene (199). Even though some lipidomic studies were conducted in animal models that mimic human exposure to toxicants, the data derived from such studies provide clues about the different classes of lipids that are enriched in coronary artery plaques in humans. This information will be useful for identifying a set of lipid biomarkers in human studies (200).

## Discussion

Research of biomarkers, and their relationship to CVD onset, progression and outcomes remain largely in its infancy. Incorporating the use of biomarkers of exposure, effect, and disease susceptibility into the current lexicon of CVD, over the long term, should contribute significantly to our understanding of cardiac pathophysiology and enable more contemporary, accurate cumulative risk stratification, diagnosis, and prognosis of cardiovascular disease and injury.

## Future Directions

**Figure 4:**
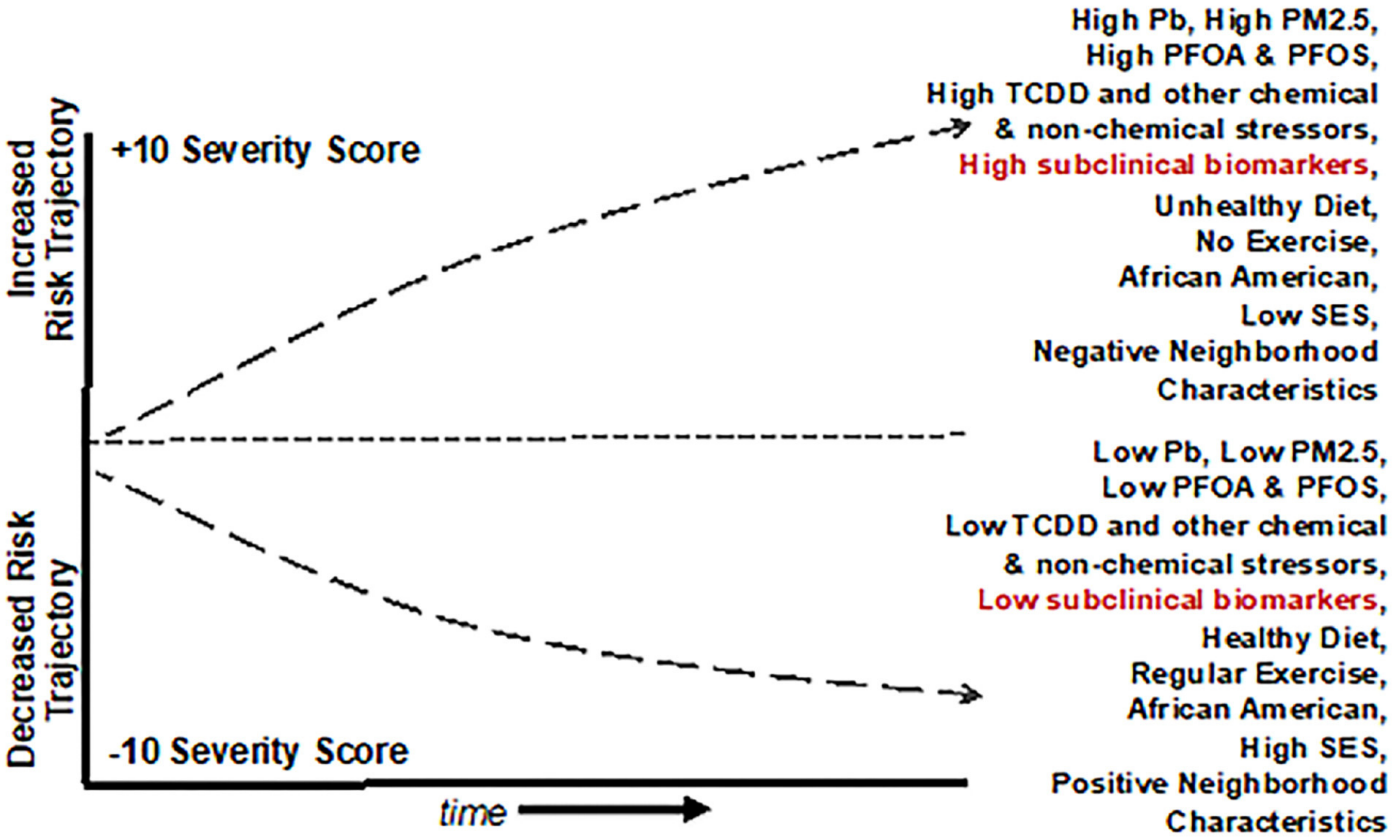
Graphical representation of a Population Level Cardiovascular Risk Trajectory Model derived from the use of *Public Health Exposome* 4.0 framework and combinatorial algorithm tool chain with input from cross-omics technologies. The model demonstrates how baseline incidence of cardiovascular risk might interact with chemical and non-chemical stressor exposures. This example shows how in African Americans, reducing PM2.5 exposures with a healthy diet, regular exercise, and socioeconomic status may combine to decrease the overall risk of CVD.

Known clinical risk factors for cardiovascular and/or cardiometabolic disease are only able to account for a fraction of the decline in the trajectory in people that have been diagnosed. Clinical variables by themselves may not be sufficient to discriminate between fast progressing and stable states of cardiovascular and cardiometabolic diseases, whereas a duality of protein markers and clinical measures, may contribute to a more robust discrimination of CVD trajectories (201). Going forward, the use of Big Data to Knowledge (BD2K), an exposome paradigm, and computational, Bayesian, and spatial temporal approaches offer tremendous promise in identifying biomarkers of subclinical, cardiovascular/cardiometabolic disease, informing novel diagnostic and treatment options, and informing public and environmental health policy (Figure 4). The purpose of such studies will be to determine if biomarkers of exposure, effect, or disease susceptibility might enhance prediction of future disease trajectories and provide earlier opportunities for intervention.

Sequencing and alignment of BD2K technologies in concert with large, novel, datasets that are harmonized within the context of multiple and longitudinal OMICS data and exposures from the natural, built, physical, and social environment holds great promise for the robust identification of cardiovascular/cardio-metabolic subclinical risk markers and our ability to discriminate cardio-metabolic trajectories in early stage patients with sub-clinical risk factors (201–203). They also are likely to provide new opportunities for completing exposures pathways, from source of exposure in the external environment to disease outcome, to population-level disparities. An exposomics approach should allow us to identify how and why people who live or spend time in certain communities experience disparate rates of CVD and other diseases. Implications of this are that it should also allow us to identify locations of place based environmental stressors associated with CVD and other diseases, and initiate actions to mitigate or protect individuals from adverse effects. Within the next half-decade, cross-omics technologies will afford opportunities to identify biomarkers for refining sub-clinical diagnosis among vulnerable subsets of patients and translate findings into clinical and policy interventions (204).

One emerging area of inquiry pertaining to CVD is integrated omics, that could immensely benefit from the recent developments in systems biology. The concept of integrated omics, also referred to as multi-omics, poly-omics, trans-omics and pan-omics includes integration of multi-dimensional omics data to provide a holistic assessment of a disease in question (205). This is an exciting approach to embrace because the etiology of some diseases is complicated as there are several causative factors. Additionally, applying one set of omics data may provide correlative information or associations resulting from reactive processes, while the causative processes go unaddressed (206, 207). Therefore, adopting a multi-omics approach is beneficial as it provides mechanistic insights into some complex diseases, which develop over time as a result of gene X environment interactions. In regard to CVD, there are some multi-omics studies, which were summarized by Leon-Mimila et al. (208). The subjects in these studies included patients suffering from various type of CVD such as coronary artery disease, dilated cardiomyopathy, congestive heart failure, and participants from the Framingham Heart Study etc. The omics strategies employed included various combinations of genomics, transcriptomics, epigenomics lipidomics, and proteomics.

The advantages notwithstanding, integrated omics approach has its own set of challenges. Each omics data set has its own complexity and completeness. Added to this, the quality of output from each analytical platform, lack of standard nomenclature, heterogeneity of data, handling large data volumes, data archiving and sharing with public are other issues (205). The recent developments in bioinformatics and big data (the BD2K technologies mentioned above) could resolve most of these issues. While the multi-omics approaches are promising to probe into the causative factors of CVD, more studies are needed to exploit the full potential of metabolomics and lipidomics in combination with other omics technologies for the identification of novel biomarkers that could be used in clinical settings and to inform environmental policy.

Another research area that deserves mention in biomarker development and deployment for assessing CVD risk from an integrated omics perspective is the microbiome. Gut microbiota has been implicated in development of cardiovascular disease. The microbiota interferes with the host metabolic pathways, resulting in obesity and insulin resistance, which increase the risk for cardiometabolic disorders [reviewed in (209) including atherosclerosis (210)]. Studies conducted on patients with coronary artery disease (CAD) showed differences in composition of gut microbial community and disease sub-types. Aside from the composition of gut microbes, the metabolites generated by these microbes also showed a significant association with severity of CAD (211). Additionally, metabolomic studies done with gut- and serum microbiome of atherosclerotic patients revealed that microbes play an important role in the progression of atherosclerosis (212). The studies of Liu et al. (211) and Kappel et al. (212) clearly indicated that the metabolites produced by the gut microbes could serve as prognostic markers for CVD risk. From the standpoint of health disparities, microbiome plays a key role as it influences the biological processes that are shaped by social and environmental factors. Integration of these processes and factors and translating the findings to communities is a major challenge, but not insurmountable (213). Therefore, in the context of obtaining a holistic view of human phenotypes and disease, incorporation of microbiome in integrative omics is beneficial to gain a thorough understanding of the disease (81).

## Limitations

One major limitation with biomarker studies is that a majority of these studies have not yet been validated. In studies involving large cohorts and multi-dimensional high throughput omics studies, reproducibility, and false positive results pose an issue (212). Typically, an ideal biomarker has to fulfill three criteria of validity, which include content validity (the extent to which a biomarker represents the biological phenomenon studied), construct validity (disease characteristics and manifestations), and criterion validity (the degree to which the said biomarker correlates with the disease) to be considered as a robust one (214). Going by these criteria, we do not yet know how many of the biomarker studies could pass the muster. Additionally, the likelihood of categorizing biomarkers as biomarkers of exposure or susceptibility by some investigators may be interpreted by others as biomarkers of effect (disease outcome). In other words, the designation of biomarkers as prognostic or diagnostic ones remain blurred (214, 215). In clinical settings, biomarker validation requires concordance between biological and clinical endpoints, disease diagnosis and disease staging etc. to enhance the utility of these markers in therapeutic applications (215). From a health disparities standpoint, there is a dearth of information on specific omics-related studies in African-American, Hispanic and Native American populations. The lack of information on validation details could be one of the key reasons why the omics approaches have not yet moved into clinic.

## Author Contributions

PJ conceived the idea of the review and he provided the conceptual framework, discussed the various issues involved in tying the exposure science to disease induction, and emphasized the relationship between exposome and cardiovascular disease. DH, AR, and M-AS contributed to the traditional and the emerging omics-based biomarker sections. PJ and DH have provided narrative on current gaps in the knowledge and future directions for research in this area. All authors collected data from literature, analyzed it, performed the review, discussed the feedback regularly, read the various iterations of the manuscript draft, contributed to its revision, and re-read and approved the final and submitted version.

## Conflict of Interest

The authors declare that the research was conducted in the absence of any commercial or financial relationships that could be construed as a potential conflict of interest.
